# Modified Sick Neonatal Score (MSNS): A Novel Neonatal Disease Severity Scoring System for Resource-Limited Settings

**DOI:** 10.1155/2019/9059073

**Published:** 2019-05-09

**Authors:** K. P. Mansoor, S. R. Ravikiran, Vaman Kulkarni, Kiran Baliga, Suchetha Rao, Kamalakshi G. Bhat, B. Shantharam Baliga, Nutan Kamath

**Affiliations:** ^1^Department of Pediatrics, Kasturba Medical College, Manipal Academy of Higher Education, Mangalore, Karnataka 575001, India; ^2^Department of Community Medicine, Kasturba Medical College, Manipal Academy of Higher Education, Mangalore, Karnataka 575001, India

## Abstract

Neonatal disease severity scoring systems are needed to make standardized comparison between performances of different units and to give prognostic information to parents of individual babies admitted. Existing scoring systems are unsuitable for resource-limited settings which lack investigations like pH, pO_2_/FiO_2_ ratio, and base excess. This study was planned to evaluate Modified Sick Neonatal Score (MSNS), a novel neonatal disease severity score designed for resource-constrained settings. It was a facility-based cross-sectional analytical study, conducted in the “Special Newborn Care Unit” (SNCU) of government district hospital, attached to Kasturba Medical College, Mangalore, India from November 2016 to October 2017. A convenience sample of 585 neonates was included. Disease severity was assessed immediately at admission using MSNS. MSNS had 8 parameters with 0, 1, and 2 scores for each. 41% of study population was preterm (*n*=240), and 84.1% had birth weight less than 2500 grams (*n*=492). The mean (SD) of the total MSNS scores for neonates who expired and discharged was, respectively, 8.22 (2.96) and 13.4 (2.14), a difference being statistically significant at *P* < 0.001. Expired newborns had statistically significant frequency of lower scores across each of the parameters. An optimum cutoff score of ≤10 with 80% sensitivity and 88.8% specificity in predicting mortality was obtained when the ROC curve was generated with the MSNS score as the test variable. Area under the curve was 0.913 (95% CI: 0.879–0.946). In conclusion, MSNS is a practicable disease severity score in resource-restricted settings like district SNCUs. It is for application in both term and preterm neonates. Total score ≤10 has a good sensitivity and specificity in predicting mortality of admitted neonates when used early during the course of hospitalization. MSNS could be used as a tool to compare performance of SNCUs and also enable early referral of individual cases to units with better facilities.

## 1. Introduction

Neonatal disease severity scoring systems are extensively used in neonatal intensive care units. They are used to make standardized comparison between performances of different units as the mortality observed could be adjusted to the disease severity of admitted infants. Besides, they aid in giving prognostic information to parents of individual babies being treated in the units. They also enable determining trends in outcomes over time [1].

Various scoring systems like clinical risk index for babies (CRIB), CRIB 2, Score for Neonatal Acute Physiology (SNAP), Score for Neonatal Acute Physiology-Perinatal Extension (SNAPPE), SNAP 2, SNAP-PE2, Neurobiological Risk Score (NBRS), Neonatal Mortality Prognosis Index (NMPI), and Neonatal Therapeutic Intervention Scoring System (NTISS) have been studied. CRIB score has been primarily designed for use in preterm babies and has the advantage of being easy to calculate. CRIB II score is an improved version of earlier score. SNAP is applicable to any infant, term, or preterm, admitted to the neonatal unit, and has 28 items. SNAPPE is its perinatal extension by adding birth weight, small for gestational age, and APGAR at 5 minutes. SNAP-II is the modified version with only 6 variables and hence easier to use [1]. NTISS is based on the treatment received by the infant, which is likely to vary depending on the unit policy and hence cannot be used to compare units [1, 2]. Many of the scoring systems contain variables which require investigations like pH, pO_2_/FiO_2_ ratio, and base excess [1]. These values are difficult to obtain in resource-restricted settings.

The neonatal mortality rate (NMR) in urban India is 15 and in rural India is 31 per 1000 live births [3]. There is a glaring discrepancy between the two. Special Newborn Care Units (SNCUs) being established by the government in district and subdistrict hospitals cater the rural population and have a major role in reducing the neonatal mortality rate in rural areas. There are differences in the admission profiles and outcomes in SNCUs of different states [4]. In order to compare performances of SNCUs and also to prompt early referral of individual babies with a more severe disease to centers which are better equipped, there is a need for a suitable disease severity scoring system. Hence, this study was planned to evaluate Modified Sick Neonatal Score (MSNS), a novel neonatal disease severity score designed for resource-limited settings.

## 2. Materials and Methods

This facility-based cross-sectional analytical study was conducted in the “Special Newborn Care Units” (SNCUs) of a government district hospital, attached to Kasturba Medical College, Mangalore. Institutional Ethics Committee of the medical college approved the study. Parents of neonates included were informed, and consent was obtained. Study was conducted from November 2016 to October 2017. Sample size of 565 neonates was needed assuming sensitivity and specificity of 90%, with an absolute precision of 0.035 at 95% confidence level. A convenience sample of 600 neonates was included during the study period. Neonates on ventilator support at admission and those who were discharged against medical advice were excluded. Demographic details, gestational age, birth weight, important clinical findings with investigations, and diagnosis were recorded in the semistructured proforma. All babies were followed up till discharge, and outcomes were noted. The disease severity was assessed immediately at admission and scored using Modified Sick Neonatal Score (MSNS), as depicted in Table 1. This score was essentially a modification of another validated scoring system, Sick Neonate Score (SNS) with 7 parameters, and studied among transported neonates for use in resource-restricted settings [5]. MSNS has 8 parameters, each given a score of 0 to 2. Six parameters out of the eight were adapted unmodified from SNS. Score 0 implied the worst, and score 2 implied the best possible clinical setting for each of the parameters. It does not require investigations like pH, pO_2_/FiO_2_ ratio, and base excess estimation which are similar to SNS. Scoring with respect to two more parameters, birth weight and gestational age, has been inserted in MSNS since they are very important determinants in survival of a newborn. Scoring for blood pressure which was a parameter in SNS was not included in MSNS as it requires accurate noninvasive blood pressure monitoring machines which may not be readily available at resource-restricted settings.

The collected data were coded and entered into SPSS Statistics for Windows, version 15.0 (SPSS Inc., Chicago, Ill, USA). Results were expressed as mean with standard deviation, median with interquartile ranges, and percentages using appropriate tables. The chi-square test was used to depict the association between the individual parameters and outcome. The receiver operating characteristic (ROC) curve was generated with MSNS as the test variable to predict mortality. The optimum cutoff value was obtained from the ROC curve. Sensitivity, specificity, positive predictive value, and negative predictive value were calculated for the cutoff score. The Mann–Whitney *U* test was used to compare the scores between the expired and discharged groups in each of the individual parameters.

## 3. Results

Out of 600 newborns enrolled, 15 were excluded.  Table 2 represents the baseline characteristics of the included neonates. 41% of babies were preterm, and 84.1% of the study population had birth weight less than 2500 grams. The predominant causes of admission *n* (%) were as follows: sepsis (239, 40.9), jaundice (143, 24.4), birth asphyxia (78, 13.3), and respiratory distress syndrome (62, 10.6). 34.9% (*n*=204) of newborns were outborn/referred. The mean (SD) day of life at admission for referred cases was 2.58 (2.53) with a median (IQR) of 1 (1–4). Most of the babies were referred on day 1 of life (*n*=113, 55.4%).

Table 3 presents the frequencies of scores 0, 1, and 2 for each parameter of MSNS among expired and discharged cases. The discharged neonates had higher frequency of better MSNS scores across the parameters, the differences being statistically significant.

The mean (SD) of the total MSNS scores for neonates who expired and discharged was, respectively, 8.22 (2.96) and 13.4 (2.14), the difference being statistically significant at *P* < 0.001.

Table 4 depicts the median (IQR) for MSNS parameters among expired and discharged cases. The Mann–Whitney test done shows that the differences in scores among discharged and expired groups were highly significant for all parameters except random blood sugar.

**Figure 1:**
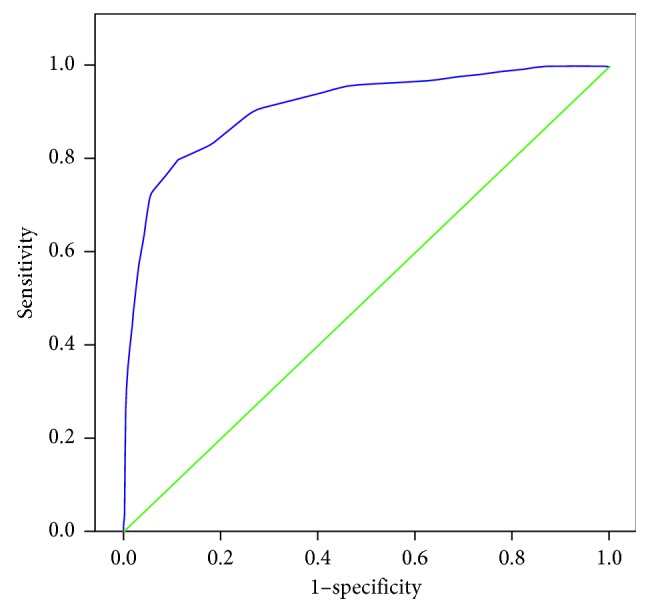
Receiver operating characteristic (ROC) curve generated with total MSNS score as the test variable to predict mortality.

The receiver operating characteristic (ROC) curve generated with MSNS as the test variable to predict mortality is shown in Figure 1. The area under the ROC curve was 0.913 (95% CI: 0.879–0.946). The optimum cutoff value obtained for prediction of mortality was 10. For a cutoff score of ≤10, sensitivity and specificity were 80% and 88.8%, respectively, in predicting mortality. Positive predictive value and negative predictive value were 58% and 95.8%, respectively. The lower the score, the higher the probability of mortality.

## 4. Discussion

In the present study, MSNS (Modified Sick Neonatal Score) parameters of 585 neonates admitted in the SNCU (Special Newborn Care Unit) were recorded. 41% of cases were preterm, and 84.1% weighed less than 2,500 grams. Overall mortality among study population was 16.2%. It has been reported that 51% of babies admitted were less than 2,500 grams, and 46% were preterm with an overall mortality rate of 10.5% across SNCUs in India [4]. The higher mortality in our study population as compared to the national average is likely to be due to differences in the disease severity among admitted newborns. The SNCU where the index study was conducted is a regional referral center for several district SNCUs.

MSNS, mean (SD) score, among expired was 8.22 (2.96) and among discharged was 13.4 (2.14). The differences were statistically significant (*P* < 0.01). Expired neonates had significantly lower MSNS scores. When individual parameters of MSNS were correlated with outcome, the lower score in each of the parameters was significantly associated with mortality.

This study done in a SNCU highlights the utility of MSNS, a novel neonatal disease severity scoring system for resource-constrained settings. MSNS had a sensitivity of 80% with specificity of 88.8% when an optimum cutoff score of ≤10 was used to predict mortality. The area under the ROC curve was 0.913 (95% CI: 0.879–0.946) which is comparable to that obtained by using SNAP-II, a widely reported neonatal disease severity score [6–10]. MSNS had a better sensitivity and specificity as compared to SNS, the original scoring system which was modified in the present study. At a cutoff score ≤8, SNS had sensitivity of 58.3% and specificity of 52.7%. However, SNS was studied only in transported babies while the present study also included inborn neonates [5].

A set of scoring systems SNAP, SNAP-II, SNAPPE, and SNAPPE-II have been found to be useful in various settings [1, 6–13]. SNAP score has 28 items in the scoring. SNAP-II score is a simpler version and has only 6 items but includes investigations like measurement of serum pH and pO_2_/FiO_2_ ratio [1]. These scoring systems are applicable for both term and preterm like MSNS but necessity of certain investigations makes SNAP score less appropriate for use in SNCUs.

CRIB and CRIB 2 scores have been studied primarily in preterm babies and needs base deficit to be scored [1, 14–16]. Several studies have reported the use of another scoring system, the NTISS which is based on treatment received by the neonate rather than measuring pathophysiological factors. However, treatments may vary depending on resources available and variations in clinical practice, and hence, scores may not necessarily represent disease severity [1, 2].

In summary, as compared to previous scoring systems, MSNS used in the present study is easy to use, has good sensitivity and specificity, could be applied early during the course of hospitalization, and could be used both in term and preterm babies. Thus, MSNS is a better suited neonatal disease severity score for SNCUs in view of their admission profile and resource availability.

As per a report by the Ministry of Health and Family Welfare, Government of India, there were 525 Special Newborn Care Units (SNCUs) all over India as of March 2015. 75% of babies were admitted belong to the underprivileged sections. Such units are established at district hospitals and subdistrict hospitals with annual delivery load more than 3000. The mortality rate among newborns admitted in SNCUs ranged from 2% to 19% in various states [4]. There is a necessity to compare performance of various SNCUs individually and state wise so as to initiate corrective measures in those units with poor performance. The comparison of mortality between the various units would be more meaningful if were to be adjusted for the disease severity scores of neonates admitted. Mortality rates adjusted for illness severity in a unit could also aid in measuring the improvement in the performance of the unit over time or following policy interventions. Besides, a disease severity score is necessary for prognostication and early referral of individual cases from basic SNCUs to SNCUs with better facilities. MSNS, the novel neonatal disease severity scoring system, suits these purposes.

The limitation of MSNS scoring is that risks like maternal diabetes, hypertension, chorioamnionitis, events during labor and delivery, adequacy of antenatal care, and antenatal steroid administration have not been factored. However, the presence of such risk factors could also be associated with a poor MSNS score. The scoring was done only once at admission, and serial scoring may have provided additional information. Besides, factors like nosocomial infections could have increased mortality among newborns with better MSNS scores at admission. These could have affected the predictive ability of the score. Furthermore, the study was a single-center study and needs extensive validation before implementation. Future studies are also needed to confirm applicability across different settings.

In conclusion, MSNS is a useful neonatal disease severity score specifically designed for use in district level SNCUs and such other resource-constrained settings. Total score of ≤10 could be used to predict mortality. It has the advantage of being easy to score with minimal resources in both term and preterm neonates when applied early during the course of hospitalization. It could be a tool to compare performance of SNCUs and could also be used for early referral of individual cases to units with better facilities.

## Figures and Tables

**Table 1:** Parameters of MSNS with scoring for each parameter.

Parameter	Score 0	Score 1	Score 2
Respiratory effort	Apnea or grunt	Tachypnea (respiratory rate >60/min) with or without retractions	Normal (respiratory rate 40–60/min)
Heart rate	Bradycardia or asystole	Tachycardia (>160/min)	Normal (100–160/min)
Axillary temperature (°C)	<36	36–36.5	36.5–37.5
Capillary refilling time (s)	>5	3–5	<3
Random blood sugar (mg/dl)	<40	40–60	>60
SpO_2_ (in room air)	<85	85–92	>92
Gestational age (in weeks)	<32 weeks	32 to 36 weeks + 6/7 days	37 weeks and above
Birth weight (kg)	<1.5	1.5–2.49	2.5 or above
Total	Maximum 16		

**Table 2:** Baseline characteristics of the included neonates.

Attribute	*N* (585)	%
*Gestational age*		
Preterm	240	41
Term	342	58.5
Postterm	3	0.5

*Birth weight*		
Normal (>2500 grams)	93	15.9
Low birth weight (1500–2500 grams)	211	36.1
Birth weight <1500 grams	281	48

*Sex*		
Male	320	54.7
Female	265	45.3

*Referral*		
Inborn	381	65.1
Referred	204	34.9

*Mode of delivery*		
Vaginal	438	74.9
Caesarean	120	20.5
Use of forceps/vacuum	27	4.6

*Outcome*		
Discharged	490	83.8
Expired	95	16.2

**Table 3:** Frequencies of scores 0, 1, and 2 for each parameter of MSNS among expired and discharged cases.

MSNS parameter	Score	Discharged, *n* (%)	Expired, *n* (%)	*P* value
Respiratory effort	0	54 (11)	56 (58.9)	<0.001
	1	87 (17.8)	16 (16.8)	
	2	349 (71.2)	23 (24.2)	

Heart rate	0	7 (1.4)	20 (21.1)	<0.001
	1	45 (9.2)	21 (22.1)	
	2	438 (89.4)	54 (56.8)	

Axillary temperature	0	26 (5.3)	25 (26.3)	<0.001
	1	314 (64.1)	62 (65.3)	
	2	150 (30.6)	8 (8.4)	

Capillary refilling time	0	4 (0.8)	8 (8.4)	<0.001
	1	38 (7.8)	46 (48.4)	
	2	448 (91.4)	41 (43.2)	

Random blood sugar	0	2 (0.4)	4 (4.2)	0.003
	1	72 (14.7)	13 (13.7)	
	2	416 (84.9)	78 (82.1)	

SpO_2_ (in room air)	0	15 (3.1)	37 (38.9)	<0.001
	1	44 (9)	34 (35.8)	
	2	431 (88)	24 (25.3)	

Gestational age	0	24 (4.9)	39 (41.1)	<0.001
	1	146 (29.8)	31 (32.6)	
	2	320 (65.3)	25 (26.3)	

Birth weight	0	40 (8.2)	53 (55.8)	<0.001
	1	183 (37.3)	28 (29.5)	
	2	267 (54.5)	14 (14.7)	

**Table 4:** Median (IQR) for MSNS parameters among expired and discharged cases.

MSNS parameter	Outcome	Median (IQR)	Mann–Whitney test *Z* value	*P* value
Respiratory effort	Expired	0 (0-1)	10.04	<0.001
	Discharged	2 (1-2)		

Heart rate	Expired	2 (1-2)	8.3	<0.001
	Discharged	2 (2-2)		

Axillary temperature	Expired	1 (0-1)	6.5	<0.001
	Discharged	1 (1-2)		

CRT	Expired	1 (1-2)	11.65	<0.001
	Discharged	2 (2-2)		

Random blood sugar	Expired	2 (2-2)	0.82	0.41
	Discharged	2 (2-2)		

SpO_2_	Expired	1 (0–2)	13.85	<0.001
	Discharged	2 (2-2)		

Gestational age	Expired	1 (0–2)	8.75	<0.001
	Discharged	2 (1-2)		

Birth weight	Expired	0 (0-1)	9.81	<0.001
	Discharged	1 (1-1)		

## Data Availability

The data used to support the findings of this study are included in supplementary information xls file. The data.sav (SPSS 15) file used to support the findings is available from the corresponding author on request.

## Supplementary Materials

Supplementary Materials Data of the cases included in the study are entered in excel sheet. Coding of data are as follows: sex: 1-male, 2-female; inborn/outborn: 1-inborn, 2-outborn; outcome: 1-discharged, 2-expired; gestational age: 0-preterm, 1-term; diagnosis: 1-neonatal hyperbilirubinemia, 2-sepsis meningitis pneumonia, 3-respiratory distress syndrome, 4-birth asphyxia, 5-others; delivery mode: 1-vaginal, 2-caesarean, 3-vacuum/forceps assisted; scores for each of the 8 individual parameters-0, 1, or 2; total score maximum of 16.
